# Care and referral patterns in a large, dedicated nurse-led atrial fibrillation outpatient clinic

**DOI:** 10.1007/s12471-021-01651-x

**Published:** 2021-12-17

**Authors:** F. R. Piersma, J. Neefs, W. R. Berger, N. W. E. van den Berg, R. Wesselink, S. P. J. Krul, J. R. de Groot

**Affiliations:** Department of Cardiology, Heart Centre, Amsterdam University Medical Centres, University of Amsterdam, Amsterdam, The Netherlands

**Keywords:** Atrial fibrillation, Nurse practitioner, Referral, Outpatient clinic

## Abstract

**Introduction:**

Atrial fibrillation (AF) is the most common arrhythmia and imposes a high burden on the healthcare system. A nurse-led AF outpatient clinic may alleviate the burden on the cardiology outpatient clinic by triaging patients who need care by a cardiologist or general practitioner (GP). However, care and referral patterns after initial assessment in a nurse-led AF outpatient clinic are unknown. We examined the proportion of AF patients assessed in a nurse-led clinic without outpatient follow-up by a cardiologist.

**Methods:**

All patients with AF referred to our tertiary medical centre underwent cardiac work-up in the nurse-led AF outpatient clinic and were prospectively followed. Data on patient characteristics, rhythm monitoring and echocardiography were collected and described. Odds ratio (OR) for continuing care in the nurse-led AF outpatient clinic was calculated.

**Results:**

From 2014 to 2018, 478 consecutive individual patients were referred to the nurse-led AF outpatient clinic. After the initial cardiac work-up, 139 patients (29.1%) remained under nurse-led care and 121 (25.3%) were referred to a cardiologist and 218 (45.6%) to a GP. Patients who remained under nurse-led care were significantly younger, were more symptomatic, more often had paroxysmal AF and had less comorbidities than the other two groups. After multivariable testing, CHA_2_DS_2_-VASc score ≥ 2 was associated with discontinued nurse-led care (OR 0.57, 95% confidence interval 0.34–0.95).

**Conclusion:**

After initial cardiac assessment in the nurse-led outpatient clinic, about half of the newly referred AF patients were referred back to their GP. This strategy may reduce the burden of AF patients on secondary or tertiary cardiology outpatient clinics.

**Supplementary Information:**

The online version of this article (10.1007/s12471-021-01651-x) contains supplementary material, which is available to authorized users.

## What’s new?

- With a comprehensive, standardised initial evaluation by a dedicated atrial fibrillation (AF) nurse practitioner, patients can be triaged to a cardiologist, a general practitioner or nurse-led care.
- In this study, most patients suffered from AF without significant other comorbidities.
- Most newly referred AF patients remained under nurse-led care or were referred back to their general practitioner, while only a minority of patients needed a consultation with a cardiologist.
- A dedicated nurse-led AF clinic may lead to a reduced burden of AF on the healthcare system.

## Introduction

Atrial fibrillation (AF) is the most common arrhythmia and may cause a wide range of symptoms. In 2019, approximately 360,000 patients suffered from AF in the Netherlands [1]. The prevalence is 0.4–1% in the general population but 8% in patients older than 80 years [2]. Because of the aging population, the number of cases of diagnosed AF is expected to be increased by a factor of 2.5 by 2050. On average, patients with AF visit a hospital eight to ten times a year [3]. The large (and increasing) number of patients with frequent hospital visits and admissions imposes a growing burden on our healthcare system and thus takes a considerable part of the total healthcare budget [4].

In 2012, Hendriks et al. reported that an AF outpatient clinic led by a dedicated nurse practitioner is associated with less morbidity and less mortality than usual care by the cardiologist [5]. This is explained by a more patient-tailored approach in the nurse-led outpatient clinic [6]. Patients with knowledge and understanding of AF report fewer symptoms, use more effective coping strategies and have fewer emotions related to AF [7]. Patients with more knowledge of their medication report better mental health than those who have less knowledge about their medication and diagnosis [8]. Nurse-led AF outpatient clinics are more cost effective and have shorter waiting times and fewer hospitalisation and emergency department visits than standard outpatient clinics [9].

In our tertiary academic hospital, we founded a nurse-led AF outpatient clinic in 2014 to improve AF care and reduce the burden of AF in the cardiologist’s outpatient clinic. The main objective of this prospective cohort study was to describe the care and referral patterns of patients after triage by a dedicated nurse practitioner. In particular, we were interested in the proportion of AF patients who were assessed and treated in the nurse-led clinic without outpatient follow-up by a cardiologist. We identify patient specific characteristics associated with continuing care in the nurse-led AF outpatient clinic or referral to the clinic of a cardiologist or a general practitioner (GP).

## Methods

### Study design

This single-centre, prospective cohort study was conducted in the Amsterdam University Medical Centre, a tertiary academic centre, in Amsterdam, the Netherlands from 2014 to 2018. Patients were eligible for inclusion if they were diagnosed with AF (paroxysmal or persistent) as confirmed with electrocardiography (ECG). Postoperative AF was diagnosed in patients with a new diagnosis of AF within 30 days of operation. The study was conducted according to the World Medical Association Declaration of Helsinki.

### Outpatient clinic workflow

**Fig. 1:**
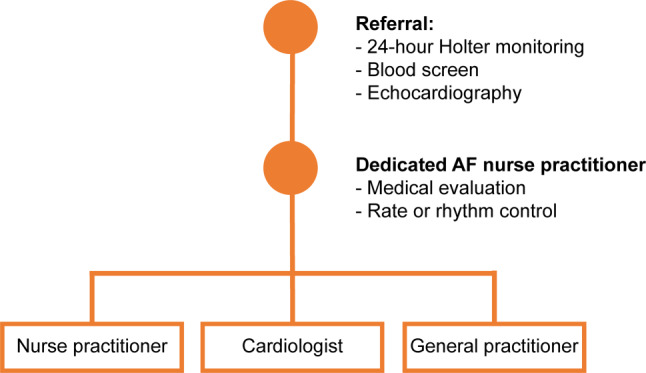
Workflow of nurse-led atrial fibrillation (*AF*) outpatient clinic

Consecutive patients underwent detailed cardiac work-up comprising registration of demographic characteristics, medical history, medication history and current prescription, 24-hour Holter rhythm monitoring and echocardiography (Fig. 1). Symptoms related to AF were classified according to the European Heart Rhythm Association (EHRA) symptom classification score, in which class I indicates no symptoms and while class IV indicates disabling symptoms [10]. All patients also completed a validated AF knowledge questionnaire prior to consultation with the nurse practitioner (Atrial Fibrillation Knowledge Scale) [11]. This questionnaire consists of multiple-choice questions in four domains: AF in general, AF therapy, AF symptom recognition and AF general attitudes. The initial questionnaire consisted of 11 questions; however, due to the current policy to prescribe a non-vitamin K antagonist oral coagulant (NOAC) to all newly diagnosed AF patients, one question concerning the function of the Thrombosis Centre with regard to regular international normalized ratio (INR) control in the Netherlands was no longer relevant.

Patients came in for an initial evaluation visit, after which they were either referred to a cardiologist or back to their GP or follow-up was continued at the nurse-led outpatient clinic. In principle, patients receiving class I or III antiarrhythmic drugs were not referred back to their GP. However, a few patients did not attend consecutive visits in the outpatient clinic and therefore stayed under control of their GP. In these cases, the GP was informed and advised to switch to another class of antiarrhythmic drugs. All newly referred patients were discussed by the nurse practitioner with a supervising cardiologist.

### Treatment

Anticoagulants, including NOACs, were prescribed to all patients with CHA_2_DS_2-_VASc score ≥ 1 (unless solely based on female sex), irrespective of the (presumed) absence of AF after ablation or the exclusion or closure of the left atrial appendage, according to current guidelines [12]. Rate control comprised antiarrhythmic drugs class II and IV and digoxin, while rhythm control comprised antiarrhythmic drug class I (i.e. flecainide) and III (i.e. sotalol, amiodarone), according to current guidelines [12]. A rate or rhythm control strategy was chosen based on the patient’s symptoms and preference and in deliberation with a supervising cardiologist. In selected cases where there was doubt about the burden of symptoms, electrical cardioversion was attempted to assess relieve of symptoms during sinus rhythm.

### Management of comorbidities

Patients who remained under nurse-led care were followed up with yearly outpatient clinic visits or more frequently if deemed necessary. Patients could also directly contact the nurse practitioner by telephone or email. A clinical consultation consisted of symptom assessment, rhythm monitoring (ECG) and blood screening. When necessary, exercise ECG or cardiac imaging was performed. Furthermore, the management of comorbidities associated with AF (mainly hypertension and obesity) was addressed in the nurse-led AF outpatient clinic. If patients reported symptoms related to obstructive sleep apnoea syndrome, polysomnography was conducted.

### Statistical analysis

For the comparison of normally distributed continuous variables, an unpaired sample *t-*test was used; the results are expressed as mean ± standard deviation (SD). In case of not normally distributed continuous variables, the Mann-Whitney U test was used; the results are expressed as median with interquartile range (IQR). Categorical variables were compared with the Pearson χ^2^ test and are expressed as frequency with percentage.

Clinical parameters associated with continuing care in the nurse-led AF outpatient clinic were assessed by univariable and multivariable logistic regression models with stepwise backward selection (removal criterion *p* > 0.10). Similar assessments were made for referral to a cardiologist or a GP. The odds ratio (OR) with corresponding 95% confidence interval (CI) was calculated.

Data analysis was performed by using R version 3.3.2 for Windows (R Foundation for Statistical Computing, Vienna, Austria). A two-sided *p*-value of < 0.05 was considered to be statistically significant.

## Results

**Fig. 2:**
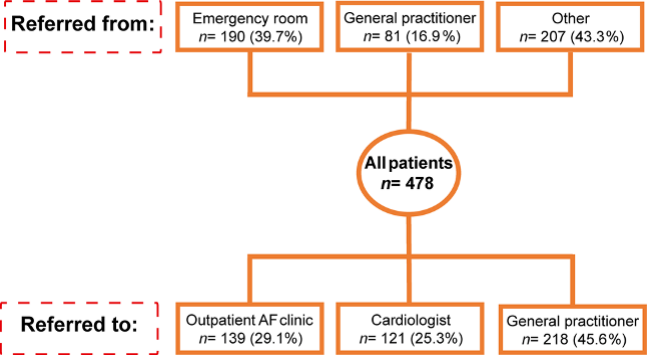
Flowchart of number of referred patients to nurse-led atrial fibrillation (*AF*) outpatient clinic and their referral after consultation with dedicated nurse practitioner

From 2014 to 2018, 478 individual patients were referred to the nurse-led AF outpatient clinic: 190 (39.7%) via the emergency room, 81 (16.9%) by a GP and 207 (43.3%) through other channels (Fig. 2).

### Patient characteristics

Patient characteristics are displayed in Tab. 1. After initial cardiac assessment, 139 patients (29.1%) remained under nurse-led care and 121 (25.3%) were referred to a cardiologist and 218 (45.6%) to a GP. Notably, patients who remained under nurse-led care were significantly younger and had less comorbidities than the other two groups. They more often had paroxysmal AF as their presenting arrhythmia. On the other hand, patients who remained under control of the nurse practitioner suffered more often from AF symptoms (higher EHRA score) than those who were referred back to their GP.

**Table 1:** Patient characteristics at initial evaluation visit for total AF patient cohort and stratified by remaining under nurse-led care, referral to cardiologist or referral to general practitioner

Variable	All patients (*n* = 478)	Nurse-led AF outpatient clinic (*n* = 139)	Cardiologist (*n* = 121)	General practitioner (*n* = 218)	*P*-value^a^
Female	191 (40.0)	51 (36.7)	48 (39.7)	92 (42.2)	0.55
BMI, kg/m^2^	26 (23–29)	26 (24–29)	26 (24–30)	26 (23–29)	0.26
Age, years	67.7 ± 12.5	65.3 ± 11.8	68.8 ± 12.1	68.7 ± 13.0	0.02
Age 65–74 years	292 (61.1)	73 (52.5)	74 (61.2)	145 (66.5)	0.02
Age ≥ 75 years	122 (25.5)	24 (17.3)	35 (28.9)	63 (28.9)	0.03
*AF type*					0.02
– Paroxysmal	317 (66.3)	101 (72.7)	68 (56.2)	148 (67.9)	
– Persistent	161 (33.7)	38 (27.3)	53 (43.8)	70 (32.1)	
Previous myocardial infarction	26 (5.4)	7 (5.0)	8 (6.6)	11 (5.0)	0.80
Previous PCI	32 (6.7)	9 (6.5)	10 (8.3)	13 (6.0)	0.72
Hypertension	245 (51.3)	61 (43.9)	72 (59.5)	112 (51.4)	0.04
Systolic blood pressure, mm Hg	138 (125–153)	137 (125–149)	140 (127–159)	136 (124–153)	0.34
Congestive heart failure	7 (1.5)	1 (0.0)	5 (4.1)	1 (0.0)	0.02
Diabetes mellitus	55 (11.5)	10 (7.2)	11 (9.1)	34 (15.6)	0.03
Vascular disease	44 (9.2)	12 (8.6)	11 (9.1)	21 (9.6)	0.93
*CHA*_*2*_*DS*_*2*_*–VASc score*					< 0.001
– 0	60 (12.6)	24 (17.2)	11 (9.1)	25 (11.5)	
– 1	110 (23.0)	44 (31.7)	34 (28.1)	32 (14.7)	
– ≥ 2	299 (62.6)	69 (49.6)	76 (62.8)	154 (68.9)	
Previous cardiac surgery	15 (3.1)	7 (5.0)	4 (3.3)	4 (1.8)	0.24
Postoperative AF	63 (13.2)	14 (10.1)	8 (6.6)	41 (18.8)	0.003
*Previous CVA*					0.18
– AF-related	22 (4.6)	9 (6.5)	6 (5.0)	7 (3.2)	
– Not AF-related	36 (7.5)	5 (3.6)	12 (9.9)	19 (8.7)	
OSAS	67 (14.0)	22 (10.8)	23 (19.0)	22 (10.1)	0.13
Valvular disease	10 (2.1)	2 (0.0)	6 (5.0)	2 (0.0)	0.04
*Thyroid disease*					0.76
– Hyperthyroidism	16 (3.3)	2 (1.4)	4 (3.3)	8 (3.7)	
– Hypothyroidism	14 (2.9)	5 (3.6)	3 (2.5)	8 (3.7)	
Family history	111 (23.2)	37 (26.6)	29 (23.9)	45 (20.6)	0.32
*EHRA class*					0.01
– I—no	139 (29.1)	30 (21.6)	28 (23.1)	81 (37.2)	
– II—mild	103 (21.5)	34 (24.5)	33 (27.3)	36 (16.5)	
– III—severe	188 (39.3)	61 (43.9)	50 (41.3)	77 (35.3)	
– IV—disabling	32 (6.7)	9 (6.5)	9 (7.4)	14 (6.4)	
Creatinine	81 (67–95)	80 (67–91)	84 (71–96)	80 (66–103)	0.32
ProBNP	935 (133–1312)	449 (119–878)	848 (198–1312)	414 (113–1372)	0.52
CRP	4.3 (1.4–13)	2.7 (0.9–9.1)	2.1 (1.1–6.8)	5.9 (2.2–27.4)	0.02

Data are *n* (%), median (interquartile range) or mean ± standard deviation

*AF* atrial fibrillation, *BMI* body mass index, *CRP* C-reactive protein, *CVA* cerebrovascular accident, *eGFR* estimated glomerular filtration rate, *EHRA class* European Heart Rhythm Association symptom classification, *ProBNP* pro-brain natriuretic peptide, *OSAS* obstructive sleep apnoea syndrome, *PCI* percutaneous coronary intervention

^a^ *P*-value for difference between referral groups

At the initial evaluation visit, 6 patients (4.3%) who remained under nurse-led care were prescribed class 1c antiarrhythmic drugs and 11 patients (7.9%) received a class III antiarrhythmic drug prescription (Tab. 2). Oral anticoagulants were prescribed to 328 patients (68.6%), of whom 249 (75.9%) received a NOAC prescription. NOACs were most frequently prescribed to patients under nurse-led care (65.5%) as opposed to 55.4% who were referred to a cardiologist and 41.7% who were referred to a GP (*p* = 0.004) (Tab. 3).

**Table 2:** Distribution of antiarrhythmic drug prescription to AF patients at initial evaluation visit stratified by remaining under nurse-led care, referral to cardiologist or referral to general practitioner

Antiarrhythmic drug class	Nurse-led AF outpatient clinic (*n* = 139)	Cardiologist (*n* = 121)	General practitioner (*n* = 218)	*P*-value
Class 1c	6 (4.3)	7 (5.8)	5 (2.3)	0.25
Class II	75 (54.0)	62 (51.2)	118 (54.1)	0.87
Class III	11 (7.9)	9 (7.4)	5 (2.3)	0.03
Class IV	3 (2.2)	7 (5.8)	5 (2.3)	0.15
Digoxin	17 (12.2)	14 (11.5)	31 (14.2)	0.77

Data are *n* (%)

*AF* atrial fibrillation

**Table 3:** Anticoagulation therapy after triage stratified for remaining under nurse-led care, referral to cardiologist or referral to general practititioner

Variable	Nurse-led AF outpatient clinic (*n* = 139)	Cardiologist (*n* = 121)	General practitioner (*n* = 218)	*P*-value
*Anticoagulant*				< 0.001*
– Vitamin K antagonist	10 (7.2)	21 (17.4)	48 (22.0)	
– Non-vitamin K antagonist	91 (65.5)	67 (55.4)	91 (41.7)	

Data are *n* (%)

*AF* atrial fibrillation

* *P*-value refers to ratio between patients receiving vitamin K antagonists those receiving non-vitamin K antagonists per category

### Clinical factors associated with type of continuing care

In a univariable analysis, age under 75 years and CHA_2_DS_2_-VASc score < 2 were associated with remaining under nurse-led care. Indeed, of the patients under nurse-led care, 115 (82.7%) were < 75 years, whereas 241 patients (71.1%) referred to a cardiologist of GP were younger than 75 years. Furthermore, 69 patients (49.6%) under nurse-led care had a CHA_2_DS_2_-VASc score ≥ 2, while this was true for 230 patients (67.8%) referred to a cardiologist or GP. After multivariable testing, only CHA_2_DS_2_-VASc score ≥ 2 was associated with not remaining under nurse-led care (OR 0.57, 95% CI 0.34–0.95 for remaining under nurse-led care) (see Fig. S1a in Electronic Supplementary Material).

Persistent AF, congestive heart failure and presence of valvular disease were associated with referral to a cardiologist in a univariable analysis. Persistent AF was seen in 53 patients (43.8%) referred to a cardiologist and in 108 patients (30.3%) who remained under nurse-led care or who were referred to a GP. Furthermore, 5 (4.1%) of the patients referred to a cardiologist had heart failure compared with 2 patients (0.6%) who remained under nurse-led care or were referred to a GP. Significant valvular disease was present in 6 patients (5.0%) referred to a cardiologist and in 4 patients (1.1%) who remained under nurse-led care or were referred to a GP. After multivariable testing, heart failure (OR 6.43, 95% CI 1.34–45.85) and valvular disease (OR 4.48, 95% CI 1.23–18.11) were strongly associated with referral to a cardiologist (see Fig. S1b in Electronic Supplementary Material).

Similarly, CHA_2_DS_2_-VASc score ≥ 2, postoperative AF and presence of diabetes mellitus were associated with referral back to a GP in a univariable analysis. Of the patients who were referred to a GP, 41 (18.8%) had postoperative AF compared with 22 patients (8.5%) who remained under nurse-led care or who were referred to a cardiologist. In addition, diabetes mellitus was present in 34 patients (15.6%) who were referred to a GP and in 21 patients (8.1%) who remained under nurse-led care or who were referred to a cardiologist. After multivariable testing, postoperative AF (OR 2.02, 95% CI 1.21–4.07) and CHA_2_DS_2_-VASc score ≥ 2 (OR 1.92, 95% CI 1.26–2.93) were associated with referral back to a GP (see Fig. S1c in Electronic Supplementary Material).

Since patients with postoperative AF may possess different characteristics than those without postoperative AF, a subanalysis was performed. This resulted in similar outcomes, although persistent AF was a stronger risk factor for referral to a cardiologist in patients without postoperative AF (data not shown).

### Questionnaire on AF knowledge

In total, 417 patients (87.2%) filled out the questionnaire before the initial evaluation visit, of whom 327 (78.4%) completed all 10 questions. Patients scored differently in the four domains: (1) AF general attitudes domain (89.1% correct answers), (2) AF symptom recognition (79.8%), (3) AF therapy (75.4%) and (4) AF in general (49.5%) (data not shown).

### Management of sleep apnoea

Obstructive sleep apnoea syndrome was previously diagnosed in 24 patients (5.0%). However, of the remaining 454 patients, 94 (20.7%) reported symptoms related to sleep apnoea, of whom 72 (76.6%) underwent polysomnography. This resulted in newly diagnosed sleep apnoea in 45 patients (9.9%).

## Discussion

After an initial evaluation visit with the AF nurse practitioner, a quarter of the newly referred AF patients remained under nurse-led care and almost half could be referred back to their GP. Only a minority of the patients needed a consultation with a cardiologist. In general, patients with multiple cardiovascular comorbidities were referred to a cardiologist, while patients with postoperative AF were referred back to their GP. Most patients, however, suffered from AF without significant comorbidities.

### Fewer referrals to cardiologist

One of our initial questions was whether the nurse-led clinic would result in a reduction of the number of referrals to the general cardiologist’s outpatient clinic or if it would mainly result in redundancy and postponement of the visit to the cardiologist. We showed that with a standardised approach, most patients were referred back to their GP after evaluation by a dedicated nurse practitioner or remained under nurse-led care. One explanation for this finding is that most AF patients, even the (very) elderly, have a limited number of cardiovascular comorbidities. The most frequently encountered comorbidities were hypertension and obesity, which are chronic conditions that, in general, can be treated in accordance with established protocols [13]. We speculate that most patients with first-time AF do not need to consult a cardiologist once the arrhythmia is managed according to current guidelines.

### Risk of cardiac complications

The seminal paper by Hendriks et al. demonstrated that in a randomised setting, patients who were treated in a nurse-led setting suffered from fewer complications and had a lower mortality rate compared with usual care [5]. In particular, adherence to six guideline recommendations (i.e. oral anticoagulation when indicated, thyroid function assessment, echocardiography, and no prescription of rhythm control in asymptomatic patients, patients with a contraindication and patients with permanent AF) was more than 80% in the nurse-led outpatient clinic versus less than 40% in the usual care arm [5]. This observation has fuelled the development of a nurse-led programme that strictly follows the European Society of Cardiology guidelines and assesses all patient in a protocolled manner. Interestingly, in a multicentre study, the same authors attempted to demonstrate superiority of the nurse-led clinic [14]. The results of that study were, surprisingly, neutral, and it was shown that the lack of benefit was driven by those clinics in which there was no or only limited experience with a nurse-led clinic.

Due to the design and the time per patient available at the nurse-led clinic, more attention can be paid to the management of comorbidities. Indeed, AF is highly prevalent among those with obesity, and weight loss is associated with a considerable rate of freedom of AF [15, 16]. Although it is not easy to adjust lifestyle, many patients achieve a more favourable weight when subjected to strict programmes [17]. Also noteworthy was our finding that when asked, a significant proportion of the patients mentioned symptoms of sleep apnoea. In 63% of the patients who were subjected to polysomnography, obstructive sleep apnoea was indeed demonstrated.

### Knowledge of atrial fibrillation and economic considerations

It has been shown previously that a large proportion of patients with AF are unable to name more than two AF symptoms and that AF knowledge is better among patients under nurse-led care compared with those under usual care [18]. Improved understanding of the arrhythmia translates to fewer negative emotions and a higher health-related quality of life [7]. There is a trend towards fewer hospitalisations and presentations to the emergency room in patients visiting the nurse-led clinic compared with those receiving usual care [19]. Moreover, knowledge combined with a plan for self-management improves lifestyle in AF patients [20]. This emphasises the notion that more time and attention are needed for the care of this chronic condition and for the coordinating role of a dedicated nurse practitioner.

The improvements associated with a nurse-led AF outpatient clinic may lead to a reduction in the burden of AF on the healthcare system and consequently a reduction in healthcare costs. The current high costs are expected to rise even further in an aging society with more subjects burdened with chronic disease, which calls for redesigning our healthcare system [6]. The costs of outpatient care are driven by diagnostic procedures, consultations and medications. Nurse-led care is associated with slightly more life-years and also more quality-adjusted life-years at a lower cost than usual care [21]. In addition, the healthcare budget is consumed by hospitalisation costs, which are reduced in patients under nurse-led care [9]. Simultaneously, an improved lifestyle is expected to have a favourable effect on a patient’s cardiovascular status as a whole.

### Study limitations

This study lacked follow-up data on cardiovascular morbidity and mortality, which could have been used to assess the consequences of a decision for referral. Importantly, the wide confidence intervals in the univariable and multivariable analyses indicated that conclusions should be drawn with caution.

## Conclusion

With comprehensive initial assessment by a dedicated AF nurse practitioner, patients can be triaged to a cardiologist, a GP or nurse-led care. A dedicated nurse-led AF clinic may lead to a reduced burden of AF on the healthcare system.

## Supplementary Information

**Fig. S1** Forest plot showing clinical parameters to predict referral to **a** nurse-led atrial fibrillation (*AF*) outpatient clinic. *OR* odds ratio, *95% CI* 95% confidence interval, *POAF* postoperative atrial fibrillation,* EHRA class* European Heart Rhythm Association symptom classification, *p p*-value

**Fig. S1** Forest plot showing clinical parameters to predict referral to **b** cardiologist. *OR* odds ratio, *95% CI* 95% confidence interval, *POAF* postoperative atrial fibrillation,* EHRA class* European Heart Rhythm Association symptom classification, *p p*-value

**Fig. S1** Forest plot showing clinical parameters to predict referral to **c** general practitioner. *OR* odds ratio, *95% CI* 95% confidence interval, *POAF* postoperative atrial fibrillation,* EHRA class* European Heart Rhythm Association symptom classification, *p p*-value
