# Supplementary figures and images for: Care and referral patterns in a large, dedicated nurse-led atrial fibrillation outpatient clinic
Source: Neth Heart J. 2021 Dec 17;30(7-8):370–6. doi: 10.1007/s12471-021-01651-x (PMC9270511; doi:10.1007/s12471-021-01651-x)

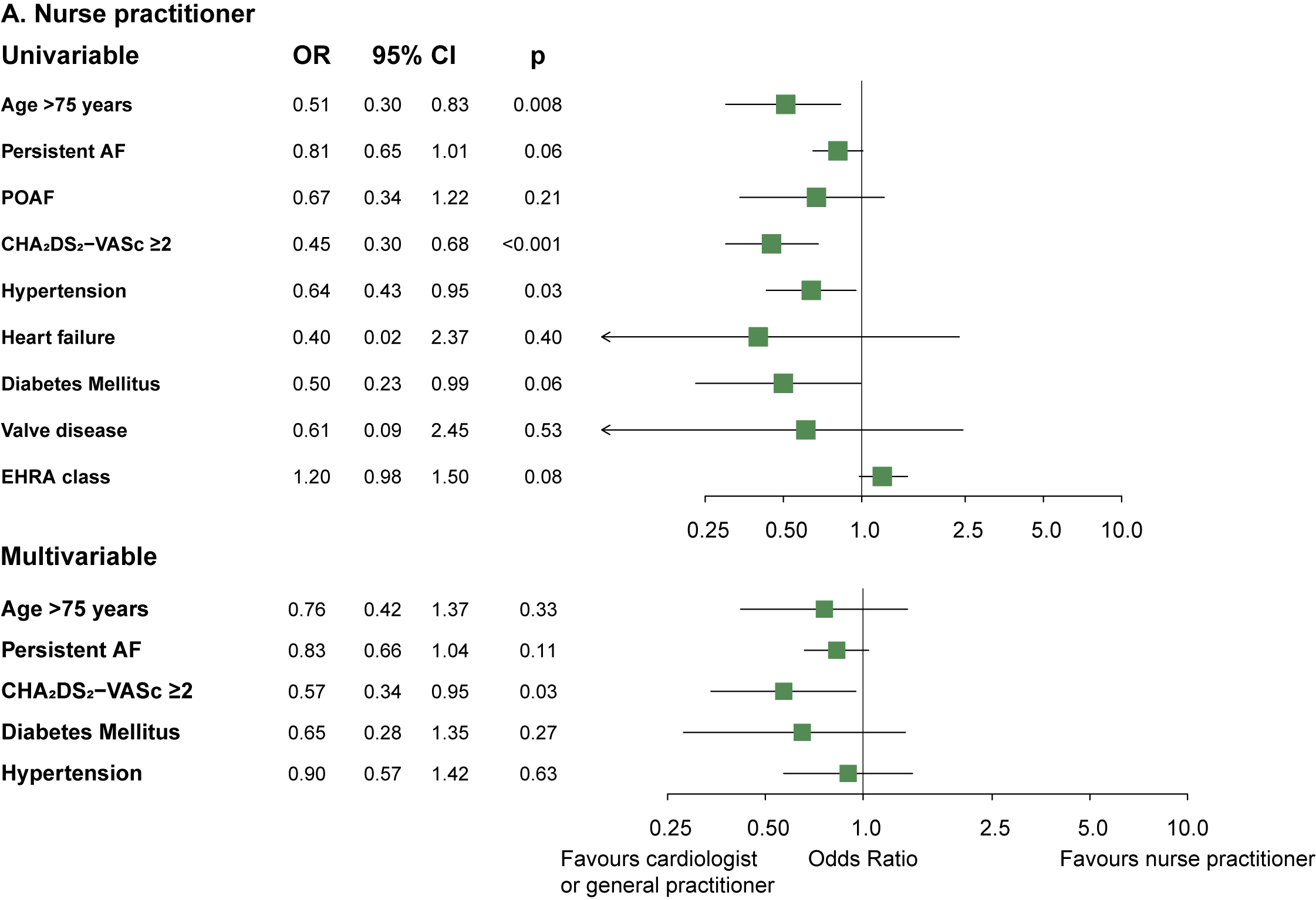

Supplement: Supplementary file 1 — Fig. S1 Forest plot showing clinical parameters to predict referral to a nurse-led atrial fibrillation (AF) outpatient clinic. OR odds ratio, 95% CI 95% confidence interval, POAF postoperative atrial fibrillation, EHRA class European Heart Rhythm Association symptom classification, p p-value [file 12471_2021_1651_MOESM1_ESM.tif]

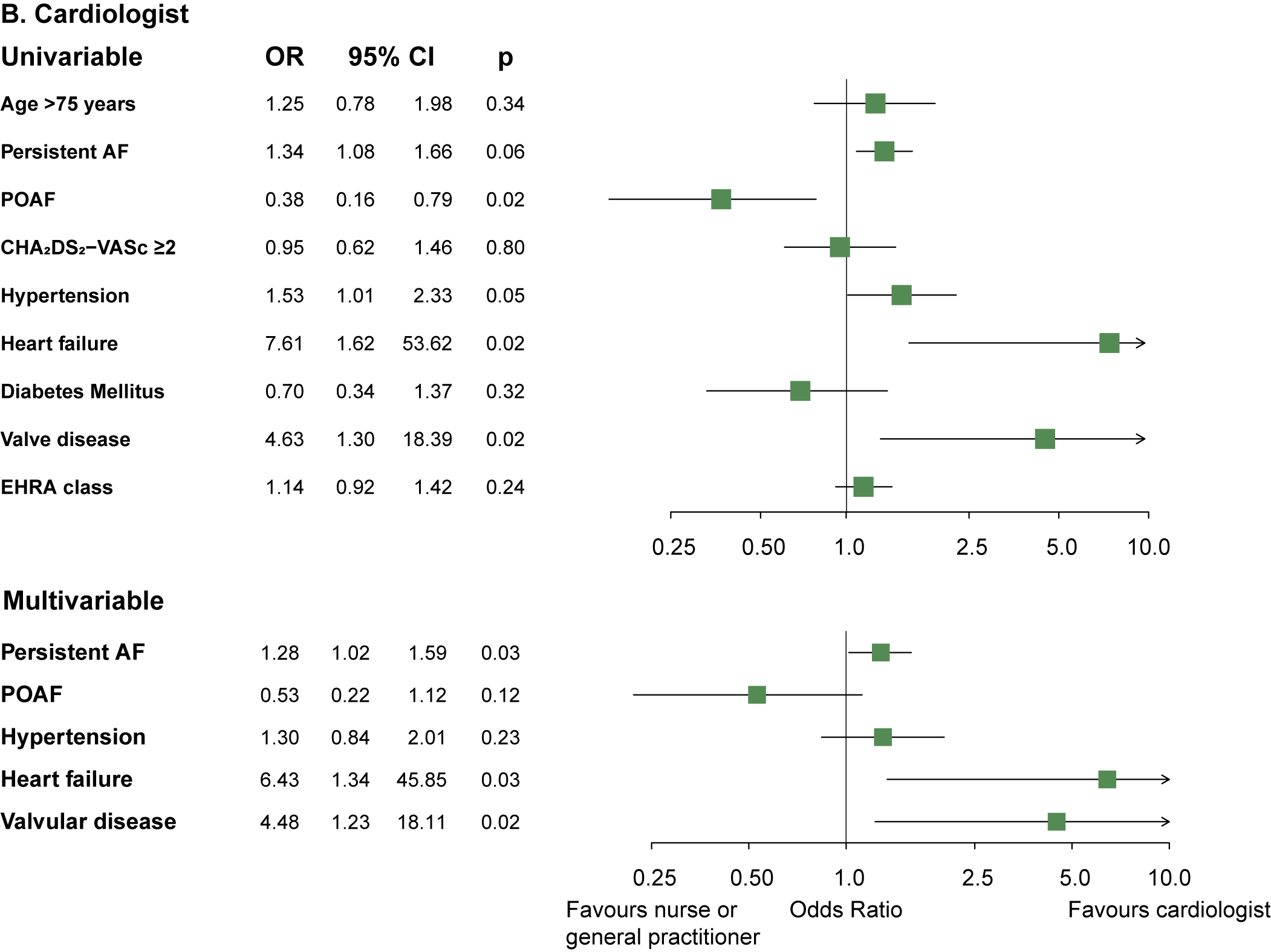

Supplement: Supplementary file 2 — Fig. S1 Forest plot showing clinical parameters to predict referral to b cardiologist. OR odds ratio, 95% CI 95% confidence interval, POAF postoperative atrial fibrillation, EHRA class European Heart Rhythm Association symptom classification, p p-value [file 12471_2021_1651_MOESM2_ESM.tif]

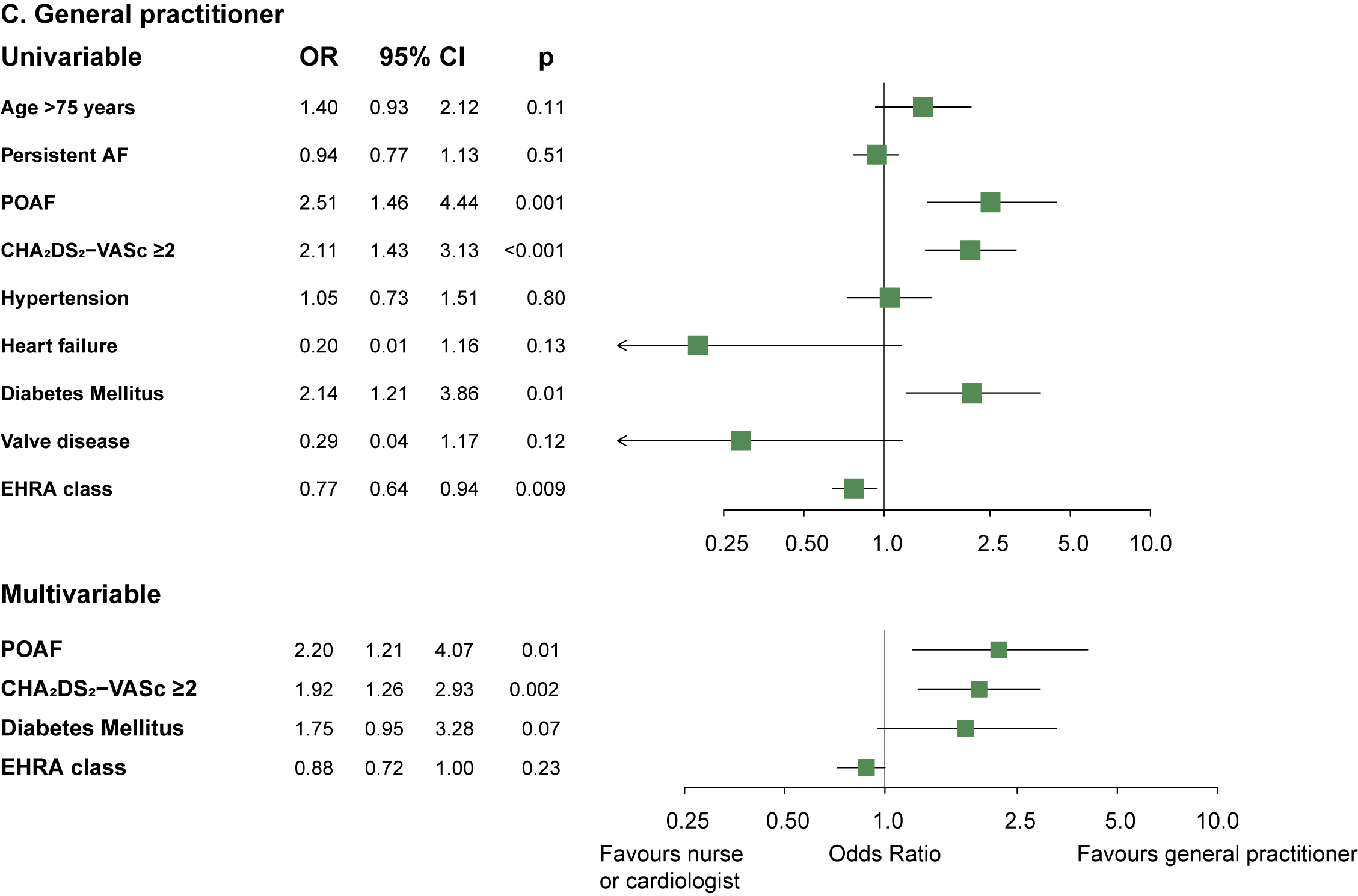

Supplement: Supplementary file 3 — Fig. S1 Forest plot showing clinical parameters to predict referral to c general practitioner. OR odds ratio, 95% CI 95% confidence interval, POAF postoperative atrial fibrillation, EHRA class European Heart Rhythm Association symptom classification, p p-value [file 12471_2021_1651_MOESM3_ESM.tif]
